# Recurrent Embolic Strokes From Prosthetic Valve Endocarditis With Negative Initial Evaluation in a Non-Toxic-Appearing Patient: A Case Report

**DOI:** 10.7759/cureus.31837

**Published:** 2022-11-23

**Authors:** Nikoloz Karazanashvili, Dena Williams

**Affiliations:** 1 Neurology, University of North Carolina at Chapel Hill, Chapel Hill, USA

**Keywords:** endocarditis, ischemic stroke, embolic stroke, infective endocarditis, infective endocarditis complications

## Abstract

Infective endocarditis (IE) is a common cause of embolic strokes. Early diagnosis and treatment are essential to decrease the risk of ischemic stroke and ensure appropriate treatment, particularly given the higher risk of hemorrhagic complications. Treatment is also essential to prevent other complications such as heart failure, perivalvular abscesses, or intracranial abscesses. Transthoracic echocardiogram (TTE) and transesophageal echocardiogram (TEE) may not show endocardial involvement in the early stages of IE and, rarely, some patients may lack systemic signs of infection, making the early detection of IE challenging. Multifocal ischemic strokes may be the initial manifestation of IE and warrant further workup directed at IE even if initial echocardiogram findings are negative. A high index of clinical suspicion and thorough history are of utmost importance.

## Introduction

Infective endocarditis (IE) is a common cause of embolic strokes and can affect the acute management of ischemic stroke, as the use of thrombolytics in embolic stroke secondary to infective endocarditis is associated with a higher risk of hemorrhagic complications and lower rates of favorable outcomes [1]. Prompt recognition and treatment of infectious endocarditis have been associated with better outcomes and decreased risk of ischemic stroke [2], underlining the importance of early diagnosis and treatment of IE. The diagnosis of IE can be challenging, as one of the major diagnostic criteria is radiographic evidence of endocardial involvement on echocardiogram, which may be negative in the early stages of IE [3]. We present a case of recurrent multifocal embolic strokes caused by prosthetic valve endocarditis without associated systemic signs of infection and no evidence of endocardial involvement on initial evaluation with a transesophageal echocardiogram.

## Case presentation

A 62-year-old female with a past medical history significant for coronary artery disease, aortic regurgitation status post replacement with bioprosthetic aortic valve (Magna Ease; Edwards Lifesciences, Irvine, CA), and a recent admission for multifocal ischemic strokes with residual left arm weakness was readmitted to the Neurology service with new-onset, right upper extremity numbness and worsening left upper extremity weakness. On examination, she was noted to have left-sided dysmetria, left arm weakness, and decreased peripheral vision in the left eye.

**Figure 1:**
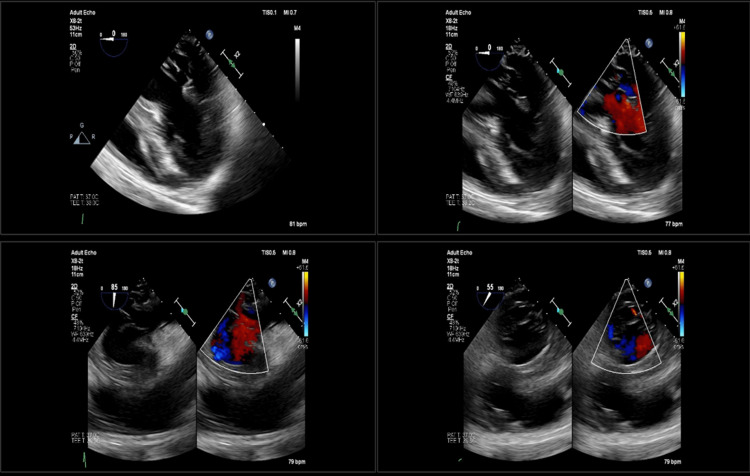
Transesophageal echocardiogram obtained during the first hospitalization without evidence of vegetation or intracardiac thrombus

She was originally admitted to the Neurology service approximately four weeks prior to this presentation with acute-onset left arm weakness and was found to have multifocal supratentorial and infratentorial infarcts. No dysmetria or visual deficits were noted at that time. Transthoracic echocardiogram (TTE) and transesophageal echocardiogram (TEE) at that time did not show evidence of vegetation or intracardiac thrombus (Figure 1). Computed tomography angiography (CTA) head and neck did not reveal significant plaques, stenosis, or dissection. There was no evidence of mycotic aneurysms. No atrial fibrillation was detected during a 48-hour telemetry monitoring.

**Figure 2:**
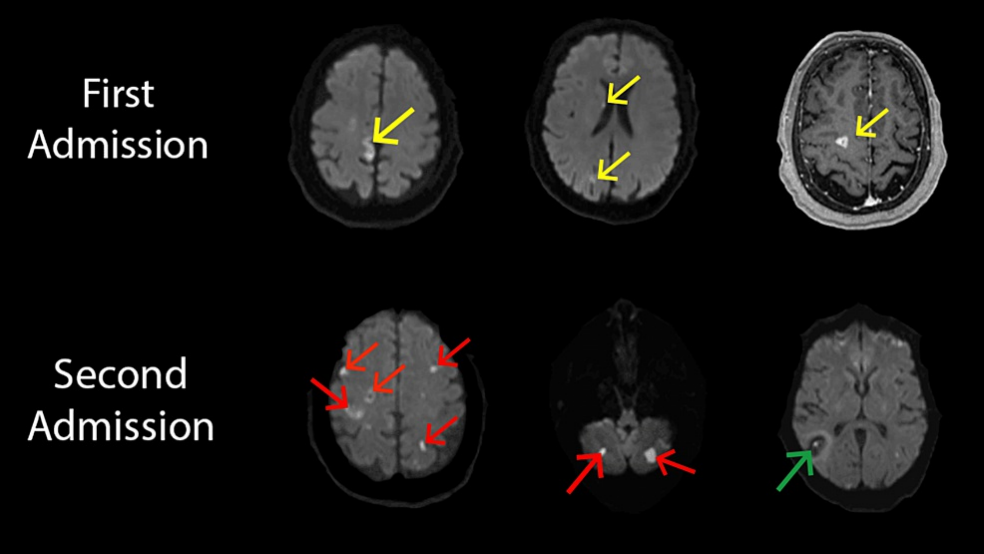
Multifocal ischemic strokes (yellow arrows) on magnetic resonance imaging (MRI) brain obtained during the first admission compared to new multifocal ischemic strokes (red arrows) as well as hemorrhagic conversion of the old parietal infarct (green arrow) on MRI from the second admission

On readmission, the patient was noted to have decreased peripheral vision in the left eye, mild weakness in the left upper extremity, and bilateral dysmetria markedly worse on the left side. She was normotensive and afebrile. Labs were remarkable for mild leukocytosis to 13.2 x 10^9^/L with a left shift. The electrocardiogram showed a new, first-degree atrioventricular block that was not present on review of telemetry from the previous hospitalization. Magnetic resonance imaging (MRI) head revealed new supratentorial and infratentorial ischemic strokes and hemorrhagic conversion of previously demonstrated right parietal ischemic stroke (Figure 2). New leukocytosis and multiple new embolic-appearing strokes in the setting of a known bioprosthetic aortic valve and otherwise negative recent stroke workup raised concern for IE. A new heart block in this setting also raised concern for developing a perivalvular abscess, hence a TEE and blood cultures were obtained despite unremarkable findings on a TEE four weeks prior and negative TTE on readmission. Notably, the patient did not have fever, chills, or any other systemic signs of bacteremia. Additionally, there was no evidence of other pathognomonic clinical signs of IE such as Janeway lesions, Osler nodes, or Roth spots. The patient tested negative for human immunodeficiency virus (HIV) and had no other immunocompromising conditions that could have explained the lack of systemic signs of infection. The patient denied intravenous drug use, recent skin trauma, or articular injections. She reported a routine dental cleaning procedure one month prior to the readmission but denied recent invasive dental procedures or tooth extraction.

**Figure 3:**
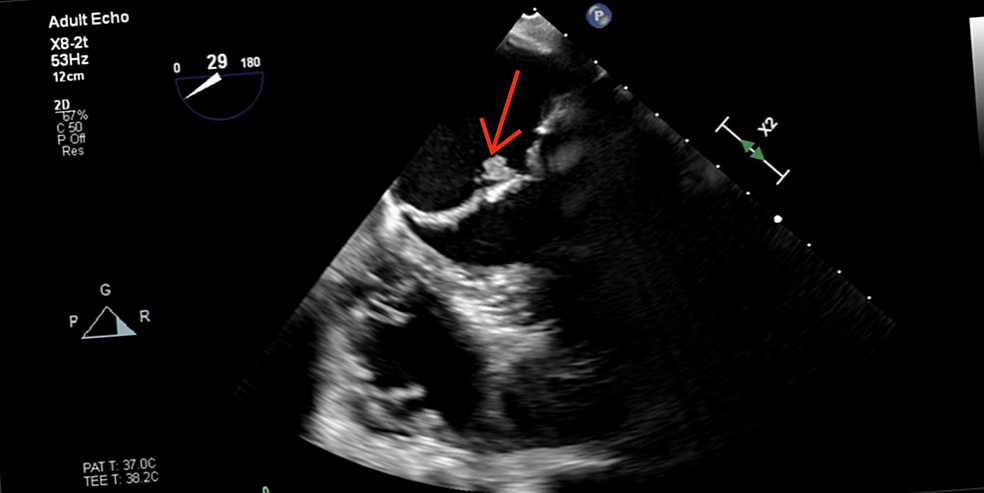
A large mobile vegetation involving the anterior mitral valve demonstrated on a repeat transesophageal echocardiogram

Blood cultures grew Staphylococcus epidermis, prompting the initiation of intravenous antibiotics. Repeat TEE revealed large vegetation involving one of the aortic prosthetic valve leaflets with associated mild aortic regurgitation, as well as vegetation involving the anterior mitral leaflet (Figure 3) and an aortic root/paravalvular abscess. The patient was treated with prolonged intravenous antibiotic therapy and eventually underwent surgical repair of the aortic valve and mitral valve and pericardial patch repair of the aortic annular abscess.

## Discussion

Radiographic evidence of endocarditis as demonstrated by transthoracic and transesophageal echocardiogram is one of two major diagnostic criteria of endocarditis [4]. It is well-known that while transthoracic echocardiogram has a good negative predictive value for ruling out endocarditis in patients with native heart valves, the diagnostic accuracy of TTE is limited in patients with prosthetic heart valves [5,6]. While the sensitivity of transesophageal echocardiogram for the evaluation of native and prosthetic valve endocarditis is markedly better than transthoracic echocardiogram, the sensitivity of TEE is not 100% and could be affected by various factors such as echogenicity and vegetation size, vegetation location, presence of prosthetic material, experience/skill of the examiner, and pretest probability of endocarditis [3]. Early recognition of IE as a potential cause of embolic strokes is clinically important in the workup and management of acute ischemic stroke, as traditional thrombolytics carry a higher risk of intracerebral hemorrhage and lower rates of favorable outcomes in patients with infective endocarditis [1,7]. Furthermore, delayed or inadequate antimicrobial therapy is associated with overall greater in-hospital mortality rates in patients with IE [7] and some studies have shown significant reductions in the risk of stroke related to IE after the initiation of appropriate antimicrobial therapy [2]. Thus, a negative transesophageal echocardiogram result should not delay the initiation of antimicrobial therapy if clinical suspicion of endocarditis remains high [8]. Repeating TEE 7-14 days after the initial negative result may reveal vegetations that were missed on the initial exam [3], better characterize complications such as perivalvular abscess, and further guide management [9].

In our case, we believe that the patient likely had endocarditis on initial presentation despite negative TEE. Multifocal strokes at that time were likely a result of septic embolism given the appearance and distribution of lesions, clinical progression, and lack of an alternative explanation despite extensive workup. It is possible that vegetations were too small to be visualized on the first TEE. Notably, at the time of initial presentation, blood cultures were not obtained as the patient was nontoxic appearing without leukocytosis and had no history of immunocompromise. Interestingly, even on readmission, the patient did not have any clinical signs of IE such as fever, chills, or evidence of embolic phenomena on physical exam. This underlines the importance of pursuing workup for IE with blood cultures and imaging even if systemic signs of infection are absent in patients with multifocal embolic-appearing strokes and risk factors for IE.

## Conclusions

Infectious endocarditis is a common cause of embolic strokes and should remain on the differential even if initial echocardiogram findings are unremarkable and systemic signs of bacteremia are absent, particularly in those with notable risk factors such as prosthetic valves. Multifocal embolic strokes may be the first clinical manifestation of IE and may necessitate a repeat TEE and blood cultures even if the initial evaluation with an echocardiogram was negative, especially if alternative sources of thromboembolism have been ruled out.
